# Effects of Weight Loss and Aerobic Exercise Training on Adi-Pose Tissue Zinc α2-Glycoprotein and Associated Genes in Obesity

**DOI:** 10.3390/cells12192366

**Published:** 2023-09-27

**Authors:** Shealinna X. Ge, Guoyan Li, Alice S. Ryan

**Affiliations:** 1 Department of Dermatology, University of Maryland School of Medicine, Baltimore, MD 21201, USA; 2 Division of Gerontology and Palliative Medicine, Department of Medicine, University of Maryland School of Medicine, 655 W Baltimore Street, Baltimore, MD 21201, USA; 3 Baltimore VA Medical Center, Geriatric Research, Education and Clinical Center (GRECC), 10 N Greene Street, Baltimore, MD 21201, USA; 4 VA Research Service, VA Maryland Health Care System, 10 N Greene Street, Baltimore, MD 21201, USA

**Keywords:** ZAG, HSL, β3AR, adipose tissue, body composition, weight loss and exercises

## Abstract

Zinc α2-glycoprotein (ZAG) has been implicated in fatty acid metabolism and utilization and is lower in obese and higher in cachexic adults compared to those of normal weight. Previous studies suggest that ZAG binds to the beta3-adrenergic receptor (β3AR) to influence fatty acid metabolism in adipose tissue by regulating hormone sensitive lipase (HSL). The purpose of this study is to investigate the effects of a six-month weight loss (WL) or aerobic exercise (AEX) intervention on adipose tissue and skeletal muscle ZAG mRNA levels and protein expression, as well as the expression of β3AR, and HSL. Abdominal adipose tissue (AB) and gluteal adipose tissue (Glut) and vastus lateralis muscle biopsies were performed before and after WL (*n* = 13) or AEX (*n* = 13). ZAG, HSL, and β3AR expressions were determined by RT-PCR, and ZAG and HSL plasma levels by ELISA. Body weight decreased by 9.69% (*p* < 0.001) in WL and did not change with AEX. Maximal oxygen consumption (VO_2_max) increased by 7.1% (*p* < 0.005) after WL and by 16.69% (*p* < 0.001) after AEX. WL significantly decreased body weight with a reduction of percentage of fat, fat mass, fat-free mass (FFM). AEX decreased percent fat and increased VO_2_max, but did not change fat mass and FFM. Abdominal ZAG and HSL mRNA levels did not change significantly after WL or AEX. There were no changes in plasma ZAG, HSL and adipose tissue β3AR mRNA levels after WL and AEX. ZAG, HSL and β3AR mRNA expressions in adipose tissue are positively associated each other. Adipose tissue abdominal and gluteal HSL are negatively associated with HOMA-IR (Homeostatic Model Assessment for Insulin Resistance), and both ZAG and HSL adipose tissue are negatively associated with fasting glucose and the glucose area under the curve. Further work is needed to elucidate the role of ZAG and HSL in the propensity for weight gain and the ability of exercise to mitigate these responses.

## 1. Introduction

Obesity and its related metabolic diseases and downstream health effects are significant sources of morbidity and mortality in the modern world [1]. A key player in the obesity epidemic is adipose tissue. Previously regarded as only for energy storage, adipose tissue has been shown to have a dynamic role in endocrine functions, energy metabolism, and physical homeostasis [2,3]. Zinc α2-glycoprotein (ZAG) is a 41 kDa soluble glycoprotein. First discovered over-expressed in the serum of patients with malignant tumors of the breast and prostate [4], ZAG was observed to be associated with adipose tissue atrophy in cachexic patients. ZAG is mainly expressed and secreted by white adipose tissue (WAT,) but is also found in brown adipose tissue (BAT), heart, liver, skeletal muscle, sweat glands, gastrointestinal tract, breast, and prostate cancer [5,6,7]. ZAG induces lipolysis and decreases body fat percentage and body weight by inhibiting lipogenic enzymes and upregulating lipolytic enzymes in adipose tissue, including adipocyte adenyl cyclase [5,7,8]. ZAG mRNA expression is lower in the adipose tissue of obese and overweight individuals compared to that of lean individuals [9]. Furthermore, plasma level of ZAG was negatively correlated with insulin and the homeostasis model assessment for insulin resistance index (HOMA-IR) [9]. In a ZAG overexpressed mice model, hormone sensitive lipase (HSL) mRNA increased in epididymal adipose tissue [8].

β3-adrenergic receptor (β3AR) belongs to the G protein coupled receptor (GPCR) family involved in a variety of cellular pathways. While mainly expressed in brown and white adipose tissue, where it functions to stimulate lipolysis, thermogenesis, and fatty acid synthesis, β3AR is also expressed in the brain, retina, myocardium, gallbladder, kidney, and urinary bladder [10,11]. β3AR is the main regulator of brown adipose tissue thermogenesis in response to cold exposure or increased dietary energy intake through activation of mitochondrial UCP-1 [12,13]. It also induces a white to brown adipose phenotypic switch in an AMP-activated protein kinase (AMPK) dependent manner. More specifically, reduced β3AR signaling decreases AMPK phosphorylation, which prevents fatty acid uptake by BAT and ultimately leads to increased body fat mass [14,15]. ZAG has been shown to bind and activate β3AR [16]. Ob/Ob mice treated with ZAG (50 μg/d orally in the drinking water) demonstrated increased protein expression of β3AR in white adipose tissue (WAT), brown adipose tissue (BAT), and gastrocnemius muscle with a reduction in glucose area under the curve during an oral glucose tolerance test [17]. 

HSL also plays a significant role in lipid metabolism and as a crucial rate-limiting enzyme for diacylglycerol catabolism. It is highly expressed in WAT and BAT, as well as expressed in skeletal and cardiac muscle, adrenal, ovaries, testis, steroidogenic cells, pancreatic β-cells, and macrophages [18]. HSL is a lipolysis-related protein that facilitates the mobilization and hydrolysis of stored fats. In the WAT of ZAG knock-out mice, p-HSL and β3AR expressions are significantly increased after ZAG-recombinant plasmids are injected through the tail vein [19]. Inhibition of HSL activity can prevent the accumulation of free fatty acids, improve insulin sensitivity and control of blood glucose in type 2 diabetes [20]. The regulation of HSL has been studied in adipose tissue, but less is known regarding HSL in plasma. 

We previously have shown in postmenopausal women that abdominal adipose tissue ZAG mRNA expression is negatively correlated with visceral adipose tissue and positively with maximal aerobic capacity (VO_2_max) [21] suggesting that central obesity and fitness play a role in ZAG adipose tissue expression. Furthermore, we were the first to demonstrate that six months of weight loss alone or combined weight loss and aerobic exercise increases, albeit not reaching statistical significance, ZAG expression in adipose tissue. Our results also demonstrated in the total group that there were inverse relationships between changes in ZAG expression and body composition and metabolism. Specifically, we observed that increased ZAG adipose tissue expression after the weight loss and combined intervention was associated with (1) loss of visceral fat and (2) reduction in fasting glucose suggesting that altering ZAG expression may be important to obesity and glucose metabolism. However, this study was conducted only in postmenopausal women, in adipose tissue only, and we could not independently test the effects of weight loss or exercise training. Therefore, we sought in this new current study to include older men, study ZAG in skeletal muscle and adipose tissue, and conduct separate interventions of weight loss and exercise training as well as test other associated genes in obesity including β3AR and HSL.

The purpose of this study is to investigate the effects of a six-month weight loss (WL) or aerobic exercise (AEX) intervention on adipose tissue and skeletal muscle ZAG mRNA levels and protein expression, the expression of β3AR and HSL, and the relationships between these genes. 

## 2. Materials and Methods

Overweight or obese adults (body mass index (BMI) of 25–50 kg/m^2^) aged 50–70 years living in and around Baltimore, Maryland and Washington District of Columbia area were eligible for study participation. Adults enrolled into the study were weight stable (<2.0 kg weight change in past 12 months) and sedentary (<20 min of structured physical activity 2 times/week). Men and women were excluded from participating in the study for the presence of heart disease, diabetes, cancer, anemia, dementia, untreated dyslipidemia, or other unstable or chronic diseases affecting the liver, lungs, or kidneys. Participants were not taking any medications for weight control. Undergoing menopause at least one year prior to participation was required for female participants. Informed consent was obtained from each person. Potential participants underwent a two-step screening process first via telephone then in person, during which each individual underwent a physical examination including a comprehensive past medical history and had a fasting blood sample drawn to obtain a blood profile. In addition, each person underwent a graded exercise treadmill test prior to study participation. The study was approved by the Institutional Review Board at the University of Maryland at Baltimore and the local Baltimore Veterans Affairs Research and Development Committee.

### 2.1. WL and AEX

Participants met with a Registered Dietitian (RD) and received instructions on maintaining a Therapeutic Lifestyle Changes (TLC) diet [22]. To maintain subject number in the weight loss classes and exercise group, participants were randomly recruited and assigned to group intervention of WL or AEX (uncontrolled two-arm intervention study). Participants in the WL group attended weekly group classes led by a RD to achieve a ~500 kcal/d hypocaloric diet. The participants maintained a food diary and were weighed weekly on a calibrated scale at class [23]. Diets were evaluated by 7-day food records using the American Diabetes Association exchange list system. The objective was to induce an approximate five to ten percent weight loss over the six months. The AEX group exercised (treadmill walking or jogging) three times per week at our research gym facility for six months. All exercise sessions were supervised by an exercise physiologist, who monitored heart rate during exercise with heart rate monitors (Polar Electro Inc., Lake Success, NY, USA). Intensity of exercise and duration progressed as previously described [23]. Blood pressure was measured prior to exercise and at the end of each exercise session for safety purposes. The progression for exercise prescription was recorded for each participant to ensure it was appropriate. Attendance was taken at both the weight loss classes for the WL group and exercise classes for the AEX group.

### 2.2. Fitness and Total Body Composition

VO_2_max was measured by indirect calorimetry (Quark, Cosmed USA, Chicago, IL, USA) during a maximal graded treadmill test where the protocol had a constant speed, and grade was increased during the test as previously described [24]. Two of three criteria were required to be met to achieve VO_2_max, including reaching 1.10 for respiratory exchange ratio, reaching greater than 90% of age-predicted maximal heart rate or having a plateau in oxygen consumption. Body weight (kg) was assessed using a calibrated scale. The participant was wearing light clothing and no shoes to obtain a true weight. Percent body fat, fat mass, lean body mass, and fat-free mass (FFM) were determined by dual-energy X-ray absorptiometry (iDXA, LUNAR Radiation Corp., Madison, WI, USA). The scans were conducted in the Baltimore Veterans Affairs department of Radiology by a certified radiologic technologist. 

### 2.3. Oral Glucose Tolerance Test (OGTT) and Homeostatic Model Assessment of Insulin Resistance (HOMA-IR)

All participants were weight stable ± 2% on the TLC diet for at least two weeks prior to testing, which were completed 24–36 h after exercise for the AEX group. Participants presented for each of these tests after a 12 h overnight fast. For the OGTT, they were given 75 g glucose following baseline sampling. Repeat samplings were completed at 30 min intervals for 2 h after ingestion of glucose. Samples were collected as previously described [23]. Plasma glucose concentrations were measured using the glucose oxidase method (2300 STAT Plus, YSI, Yellow Springs, OH, USA). Plasma insulin was measured in duplicate by radioimmunoassay (RIA) (Millipore, St. Charles, MO, USA). All samples from the same participant were assayed in the same RIA. The homeostatic model assessment of insulin resistance (HOMA-IR) was calculated using fasting insulin and fasting glucose as described [25]. 

### 2.4. Adipose Tissue Biopsies

On a different day, participants underwent two separate adipose tissue aspirations. They were provided with an eucaloric diet to consume for two days before the fat aspirations. Subcutaneous adipose tissue was aspirated under local anesthesia (0.5% xylocaine) from both the abdominal and gluteal regions using a 10 mm mini-cannula. Adipose tissue was rinsed with saline and immediately freeze-clamped. The adipose tissue was then stored at −80 °C until analysis for gene expression described below. 

### 2.5. Resting Metabolic Rate and Skeletal Muscle Biopsies

On a separate day after being provided for two days of eucaloric diet, participants underwent resting metabolic rate determination as described [26]. Briefly, resting metabolic rate (RMR) was determined by indirect calorimetry and the measurement of breath by breath gas exchange to assess the production of carbon dioxide (VCO_2_) and oxygen consumption (VO_2_) in addition to other parameters. Participants fasted for this test for 12 h prior and did not have liquids except for water (no caffeine consumption). The test was conducted for 30 min. After the completion of the RMR test, participants underwent a fasting vastus lateralis muscle biopsy [23]. Muscle samples were frozen immediately in clamps cooled in liquid nitrogen and stored @ −80 °C until RNA extraction. 

### 2.6. RNA Extraction, cDNA Synthesis, and RT-PCR

Approximately 1 g of adipose tissue was used for RNA isolation [27] and 50–80 mg of muscle was used for RNA isolation. Total RNA extraction, cDNA synthesis, and quantitative Real-Time PCR (qPCR) were performed as reported previously [28,29]. The primer/probes that were used include Thermosphere Human ZAG: assay ID Hs00426651_m1, Human HSL assay ID Hs00193510_m1, Human Beta3AR assay ID: Hs00609046_m1. The expression levels of each mRNA were normalized to 36B4 mRNA. Both the basal and insulin-stimulated muscle tissue mRNA were run in the same assay. Likewise, the abdominal and gluteal adipose tissue from each participant were quantified in the same run.

### 2.7. Plasma ZAG and HSL Detection

Participants arrived at the lab in the fasted state as defined by no ingestion of food or drink except water for the prior 12 h. Blood samples were collected in syringes and placed on ice. The blood cells were centrifuged at 4 °C within 2 h at 3000 rpm for 15 min. Using an individually wrapped sterile disposable transfer pipet, approximately 500 microliters (0.5 mL) of plasma was placed into a cryovial tube, and then stored in a −80 °C freezer until analysis. Plasma ZAG and HSL level were determined in duplicate by enzyme-linked immunosorbent assay (ELISA) assay (ZAG: Antibodies-online Inc., Pottstown, PA, USA, ABIN1979303. HSL: NOVUS, Centennial, CO, USA, NBP2-76594) according to the manufacturer’s instructions.

### 2.8. Statistical Analyses

The comparison between the changes in measures between WL and AEX were assessed using one-way ANOVA. Pearson correlational analyses were conducted to examine relationships between genes and with metabolic characteristics. Statistical significance was set at a two-tailed *p* < 0.05. SPSS (IBM SPSS Statistics 27, Armonk, NY, USA) was used for data analysis; results are expressed as mean ± SEM.

## 3. Results

### 3.1. Body Composition, Glucose Tolerance Test and Gene Expression

Subject characteristics before and after WL and AEX are depicted in Table 1. The groups were similar in age, sex, and race at baseline. There was a significant difference in the change in body weight (*p* < 0.001), fat mass (*p* < 0.001), FFM (*p* < 0.05), fasting and AUC for glucose (*p* < 0.05) between WL and AEX groups. Body weight (*p* < 0.001), FM (*p* < 0.001), FFM (*p* < 0.05), and percent fat (*p* < 0.001) decreased after WL. VO_2_max increased after both WL (*p* < 0.005) and AEX (*p* < 0.001) when expressed as mL/kg/min, but only increased after AEX when expressed in L/min (*p* < 0.001). Percent fat decreased (*p* < 0.05) after AEX, but no changes in body weight, fat mass, or FFM were observed. There was a significant decrease in fasting glucose (*p* < 0.005), fasting insulin (*p* < 0.005), glucoseAUC (*p* < 0.001), insulinAUC (*p* < 0.05) and HOMA-IR (*p* < 0.005) after WL, but no changes in RMR and fasting RQ were observed. Fasting glucose, fasting insulin, glucoseAUC, HOMA-IR, and RQ did not change after AEX. InsulinAUC decreased (*p* < 0.05) and RMR increased (*p* < 0.01) after AEX.

**Figure 1:**
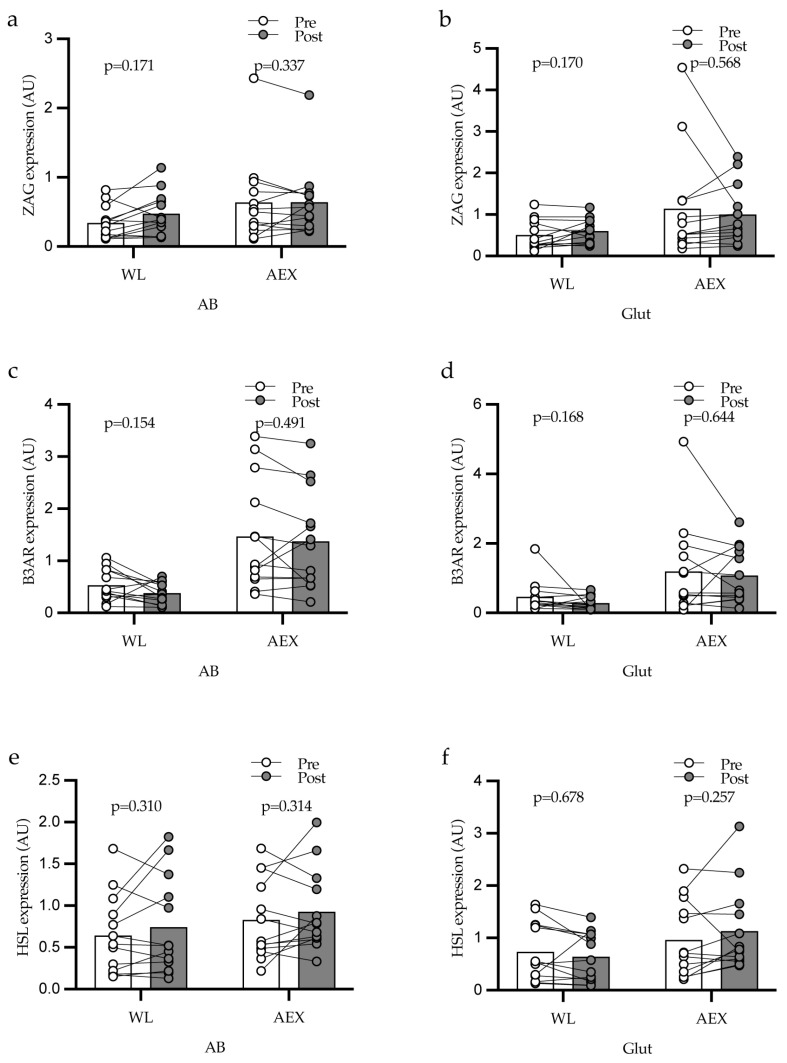
ZAG gene expression in abdominal (AB) and gluteal (Glut) adipose tissue before and after WL (**a**) and AEX (**b**). β3AR gene expression in abdominal and gluteal adipose tissue before and after WL (**c**) and AEX (**d**). HSL gene expression in abdominal and gluteal adipose tissue before and after WL (**e**) and AEX (**f**).

**Figure 2:**
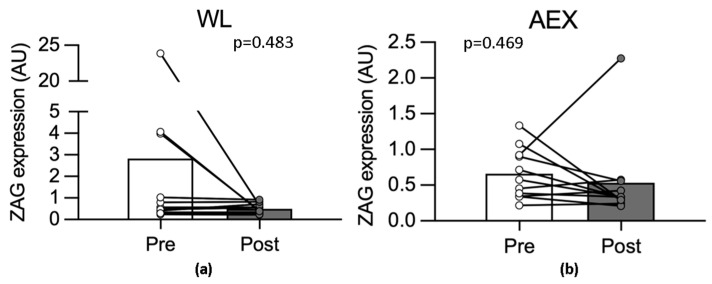
Skeletal muscle ZAG gene expression before and after WL (**a**) and AEX (**b**).

There were no significant difference in the changes in either abdominal or gluteal adipose tissue ZAG (Figure 1a,b), β3AR (Figure 1c,d), HSL (Figure 1e,f) after WL or AEX, respectively, and no significant changes within either group. Skeletal muscle ZAG did not significantly change from basal to insulin stimulation either before (1.07 ± 0.4 vs. 0.47 ± 0.06 AU) or after WL (0.50 ± 0.06 vs. 0.61 ± 0.1 AU). Likewise, there was no change from basal to insulin before (1.01 ± 0.39 vs. 0.57 ± 0.10 AU) or after AEX (0.53 ± 0.18 vs. 0.91 ± 0.31 AU). Skeletal muscle ZAG expression also did not change with either WL (Figure 2a) or AEX (Figure 2b). Plasma HSL levels did not significantly change after WL or AEX.

### 3.2. Relationships (Table 2 and Table 3)

Skeletal muscle ZAG expression correlates with both abdominal adipose tissue ZAG gene expression (r = 0.724, *p* < 0.0001) and gluteal adipose tissue ZAG expression (r = 0.589, *p* < 0.005). Skeletal muscle ZAG expression was negatively associated with fasting glucose (r = −0.582, *p* < 0.005), but no other measures of glucose metabolism or insulin resistance. In examining the relationships between adipose tissue ZAG expression and the other genes, there were strong associations with abdominal and gluteal HSL and β3AR expression. Specifically, abdominal ZAG expression was correlated with abdominal HSL (r = 0.652, *p* < 0.001) and abdominal β3AR expression (r = 0.577, *p* < 0.005). Likewise, gluteal ZAG correlated with gluteal HSL (r = 0.78, *p* < 0.001) and gluteal β3AR (r = 0.746, *p* < 0.001) expression. In addition, abdominal adipose tissue HSL is associated with gluteal adipose tissue HSL (r = 0.836, *p* < 0.001). Both abdominal and gluteal HSL were associated with abdominal and gluteal β3AR, respectively (abdominal r = 0.487, *p* = 0.01 and gluteal r = 0.508, *p* < 0.01). Plasma HSL did not correlate with HSL gene expression. 

**Figure 3:**
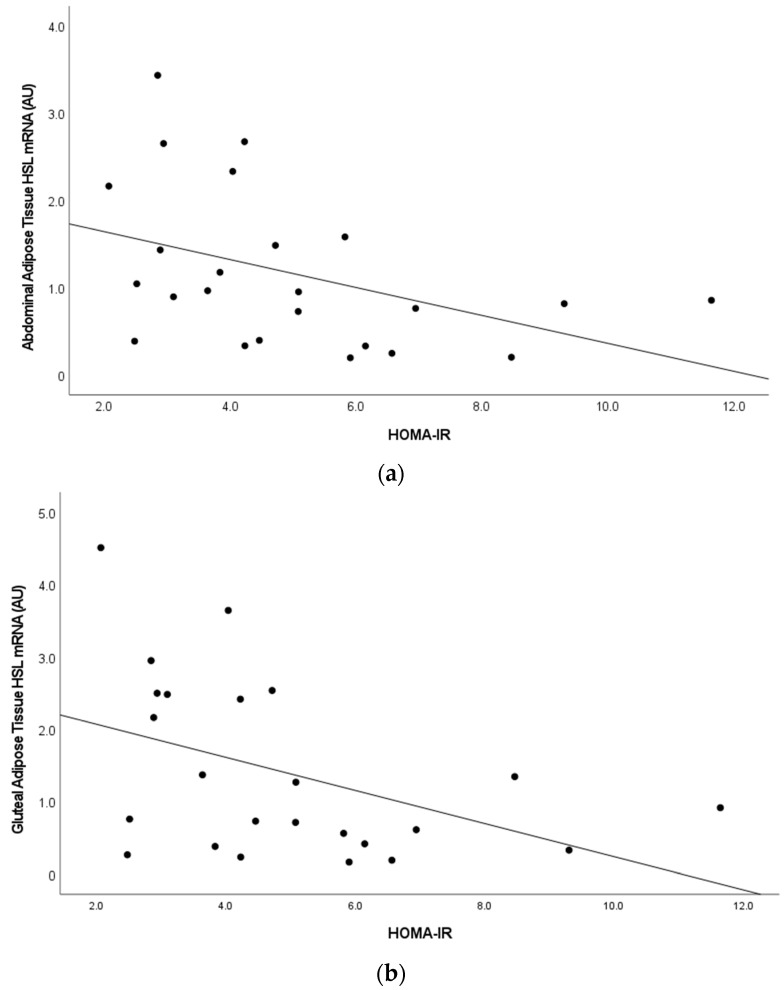
(**a,b**). Relationship between Homeostatic Model Assessment of Insulin Resistance (HOMA-IR) and abdominal adipose tissue hormone sensitive lipase gene expression (r = −0.42, *p* < 0.05) and gluteal adipose tissue hormone sensitive lipase gene expression (r = −0.44, *p* < 0.05).

In the combined sample at baseline (*n* = 26), we examined the relationships between adipose tissue ZAG, HSL, β3AR with measures of glucose metabolism (Table 3). Skeletal muscle ZAG expression only correlated with fasting glucose (r = 0.582, *p* = 0.002). Abdominal and gluteal adipose tissue ZAG expression were negatively associated with fasting glucose and glucose area under the curve (*p*’s < 0.050). Adipose tissue HSL expression was also negatively associated with fasting glucose (*p* < 0.001), glucose and insulin areas under the curve (*p*’s < 0.05), and HOMA-IR (*p*’s < 0.05, Figure 3). There was a trend for plasma ZAG to be associated with plasma HSL (r = 0.38, *p* = 0.058). Plasma ZAG was not significantly associated with measures of glucose tolerance (fasting glucose, fasting insulin, glucose and insulin areas under the curve) or HOMA-IR. Plasma HSL level correlated with fasting insulin (r = 0.487, *p* < 0.05), HOMA-IR (r = 0.461, *p* < 0.05), and RMR (r = 0.4, *p* < 0.05).

## 4. Discussion

The results of our study indicate that, despite reductions in body weight and body fat and improvements in glucose metabolism with weight loss, there was no significant change in the expression of either skeletal muscle ZAG or adipose tissue ZAG in two regions (abdominal and gluteal) with a 9.6% weight loss. Improvements in physical fitness by six months of aerobic exercise training did not result in significant changes in muscle or adipose tissue ZAG expression. Moreover, adipose expression of HSL, and β3AR did not change after WL or AEX in older men and women. 

### 4.1. ZAG: Relationships and Effects of Interventions

Expressed in a variety of tissues, ZAG plays an important role in lipid metabolism, glucose utilization, and insulin sensitivity [9,30]. ZAG mRNA expression in subcutaneous adipose tissue is reduced in overweight and obese individuals compared to lean individuals; yet there were no differences in circulating levels of ZAG or in visceral adipose tissue ZAG mRNA expression between lean, overweight, and obese subjects [9], suggesting that the origin of the adipose tissue depot may be important in ZAG levels. We examined subcutaneous ZAG mRNA expression and non-visceral fat, but did not find a relationship between subcutaneous abdominal or gluteal ZAG mRNA expression with BMI, possibly because we did not have a lean group for comparison and had only five individuals in the overweight category by BMI (three of whom the BMI was over 29 kg/m^2^). Plasma ZAG has previously been shown to be negatively associated with insulin levels and HOMA-IR in humans with normal glucose tolerance, impaired glucose tolerance (IGT), and newly diagnosed type 2 diabetes mellitus (T2DM) [31]. In contrast, we did not observe relationships between plasma ZAG with either measures of glucose tolerance (e.g., fasting glucose and insulin or areas under the curve) or insulin resistance by HOMA-IR. Likewise, plasma ZAG levels did not appear related to measures of body composition. In contrast to circulating levels of ZAG, our results indicate that ZAG expression in abdominal and gluteal tissue is negatively associated with glucose metabolism, specifically fasting glucose and two-hour glucose area under the curve. Expression of gluteal adipose tissue ZAG was also negatively associated with insulin area under the curve. In our previous study of adipose tissue ZAG expression in postmenopausal women [21], gluteal ZAG mRNA was negatively associated with fasting insulin. Together, these results could suggest that WAT-bound ZAG may be more important for normalizing blood glucose levels, and that adipose tissue ZAG is more important than soluble ZAG. 

We previously reported that ZAG expression in abdominal and gluteal adipose tissue did not significantly change after WL or combined WL and aerobic training in postmenopausal women [21]. We confirm that adipose tissue ZAG expression does not change after WL and add to the literature that it does not change in men or with aerobic training alone. We are unaware of any other published studies examining ZAG expression after WL or AEX in healthy adults. In cancer patients, subcutaneous adipose tissue ZAG mRNA expression was upregulated in the cachectic group, compared to the weight stable cancer group, and positively correlated with percent weight loss [32]. More work is needed to better understand the effects of WL and/or exercise training on ZAG levels in older adults.

### 4.2. HSL: Relationships and Effects of Interventions

HSL is a known intracellular rate-limiting enzyme that hydrolyzes triacylglycerols, diacylglycerols, monoacylglycerols, and cholesteryl esters releasing free fatty acids (FFA) from adipose tissue, and is highly expressed in adipose tissue [20]. Adipocyte lipolysis included three consecutive steps: (1) triacylglycerol (TAG) is hydrolyzed by adipose triglyceride lipase (ATGL) to generate fatty acids and di-acylglycerol (DAG); (2) DAG is catalyzed to monoacyl-glycerol (MAG) and fatty acids by HSL; and (3) MAG is hydrolyzed into one fatty acid and glycerol by monoacylglycerol lipase [20]. HSL must translocate from an aqueous cytoplasm to contact with the lipid droplet surface to hydrolyze lipids [20,33], and its lipolysis role in adipose tissue is key to influencing metabolism. Our data show that abdominal HSL expression was significantly negatively associated with BMI (r = −0.41, *p* < 0.05), indicating that HSL is reduced with higher levels of obesity. Our results confirm with others who report that ZAG mRNA expression in adipose tissue positively correlates with HSL expression [9,34]. Genetic inhibition of HSL increases insulin sensitivity in human adipocytes and mice adipose tissue through the glucose-responsive transcription factor [35]. In contrast, other studies showed that HSL mRNA and protein expression in adipose tissue were negatively correlated with fasting insulin levels and the HOMA-IR, and were higher in a subset of insulin sensitive adults than in insulin resistance participants [34]. The differences between insulin resistant and insulin sensitive subjects remained significant regardless of fat mass and after correction of mean fat cell volume or fat cell weight [34]. Our results support these studies, as participants with higher HSL mRNA levels in adipose tissue had greater insulin sensitivity (lower fasting glucose, both glucose and insulin area under the curve from the OGTT, and HOMA-IR), suggesting that HSL may also be important in regulating insulin sensitivity and maintaining metabolic balance.

Our data showed that HSL mRNA did not change with weight loss alone of almost ten percent or six months of AEX. There appears to be some inconsistencies in the literature as to the effects of WL or AEX on HSL expression. In a study examining the effect of weight reduction on adipose tissue HSL expression in obese adults between the ages of 20 to 50 years, both HSL protein and gene expression in white adipose tissue decreased after a 10-week hypocaloric diet, despite no significant changes in fasting glucose, insulin, and HOMA-IR [34]. In contrast, HSL expression significantly increased after bariatric surgery weight loss for 3–15 months in 19 obese individuals [36]. Richelsen et al. [37] also reported that HSL activity in adipose tissue was significantly enhanced after weight loss. In studies examining the effects of exercise training, moderate (*n* = 17) or high-intensity (*n* = 19) training of 12 weeks increased protein levels of HSL in subcutaneous abdominal adipose tissue [38]. Although our sample sizes are similar to these studies, it is possible that the combined negative energy balance with the exercise is responsible for the changes given that a positive energy balance or overfeeding down regulates adipose tissue pyruvate dehydrogenase kinase 4 (PDK4) and HSL mRNA expression [39]. Furthermore, the timing of measuring the expression of these genes may be important, as HSL activity increases at 3 min after exercise but does not change further at 10 min of exercise [40]. In a study that combined WL and AEX, abdominal adipose tissue HSL and pyruvate dehydrogenase kinase 4 (PKD4) gene expression were significantly up-regulated after moderate (*n* = 12) or vigorous (*n* = 12) intensity exercise training for three weeks with a dietary energy deficit of approximately 5000 kcal/week in overweight men and postmenopausal women [41]. Insulin resistance is reduced after WL intervention; the ZAG and HSL expression did not significantly change, suggesting that the improvement of insulin sensitivity after WL is not regulated by HSL or ZAG. 

Our results also indicate that neither WL nor AEX changed plasma HSL. Some studies showed that HSL, as cholesterol esterase, is present in monocytes, both before and after differentiation into macrophages [42,43]. Insulin downregulates the HSL activity in peritoneal macrophages [44]. The number of monocytes and the monocyte activation markers are significantly associated with obesity and insulin resistance [45]. Perhaps HSL in fat and plasma act via different mechanisms, which could be a driving direction for future research.

### 4.3. β3AR: Relationships and Effects of Interventions

β3AR is another regulator of lipolysis and thermogenesis in adipose tissue [12]. ZAG is highly bound to the β3AR [17]. After oral ZAG treatment, the protein expression of β3AR in white and brown adipose tissues in ob/ob mice model increased, the area under the curve during an OGTT was reduced and insulin sensitivity improved [17]. Recombinant human ZAG treatment induced body weight loss and decreased glucose level and the total area under the glucose curve [17,46]. Our data demonstrates a positive correlation between ZAG and β3AR; further, both ZAG and β3AR have the same directional associations with glucose (e.g., β3AR was also negatively correlated with fasting glucose and two-hour glucose area under the curve). 

The β3AR in adipocytes activates the extracellular signal-regulated kinases 1 and 2 (ERK) by the activation of Src kinase [47]. HSL, as a substrate of activated ERK, is activated by phosphorylating HSL at Ser(600) by activated ERK [48]. After it is activated, HSL translocates from an aqueous cytoplasm to contact with the lipid droplet surface to hydrolyze lipids [20,33]. After the ob/ob mouse was treated with ZAG (100 μg, intravenously, daily) for 15 days, body weight was reduced; HSL expression and adipose triglyceride lipase (ATGL) increased in the adipose tissue by extracellular signal-regulated kinase (ERK) pathway [49]. Thus, β3AR and HSL co-regulate the secondary step of adipocyte lipolysis. Our data showed that the adipose tissue ZAG, β3AR and HSL positively correlated with each other, and each were negatively correlated with fasting glucose and glucose tolerance, suggesting that the higher the ZAG, β3AR and HSL, the higher the fat utilization in adipose tissue in the presence of impaired glucose metabolism.

Our previous work [23,50,51] and others [52,53,54,55] has shown that weight loss, aerobic exercise training or weight loss plus aerobic exercise training can reduce body weight, body fat, and increase insulin sensitivity The current study further confirms that weight and aerobic exercise interventions improves body composition and increases insulin sensitivity.

The limitations of the study include the relatively small sample size of participants in each group, lack of a control group of individuals of normal weight, and measurement of three genes important in fat metabolism. Although muscle and adipose tissue biopsies are invasive by nature and more difficult to conduct, our study may be underpowered to detect statistical significance in the human biopsy samples. Despite the limitations, this was a carefully controlled study, long duration of interventions, and novel in the determination of ZAG in plasma, skeletal muscle and adipose tissue, and HSL in adipose tissue and plasma before and after lifestyle interventions in older men and women. A larger study is needed to confirm our results. 

## 5. Conclusions

ZAG expression in white adipose tissue of the abdominal and gluteal regions as well as in skeletal muscle did not change after six-month WL or AEX. Likewise, other associated genes, HSL, and β3AR in adipose tissue were not altered. Circulating ZAG and HSL level did not change after WL or AEX. Furthermore, ZAG, β3AR and HSL mRNA expression in adipose tissue were related to one another. Abdominal and gluteal ZAG was negatively associated with glucose metabolism. Additional work is needed to study whether ZAG and HSL play a role in body weight maintenance and the potential of exercise to modify its effects. 

## Figures and Tables

**Table 1:** Physical, metabolic characteristics and gene expression of all participants (n = 26) before and after weight loss (WL) or aerobic exercise (AEX) intervention.

	Weight Loss (WL) (*n* = 13)		Aerobic Exercise Training (AEX) (*n* = 13)	
	Pre	Post	Pre	Post
Age (yr)	63.9 ± 1.3		62.5 ± 2.4	
Sex (M/F)	6M/7F		6M/7F	
Race (C/AA)	11C/2AA		9C/4AA	
Weight (kg) ‡	101.98 ± 19.85	92.13 ± 17.60 ‡	90.85 ± 18	91.63 ± 20.39
VO_2_max (mL/kg/min)	22.49 ± 3.34	24.09 ± 4.2 **	25.11 ± 1.08	29.30 ± 1.45 ‡
VO_2_max (L/min)	2.3 ± 0.61	2.24 ± 0.0.63	2.2 ± 0.2	2.5 ± 0.2 ‡
Percent body fat	43.39 ± 7.71	39.48 ± 8.22 ‡	40.2 ± 2.5	38.9 ± 2.7 *
Fat mass (kg) ‡	43.89 ± 2.72	36.61 ± 2.65 ‡	37.6 ± 3.7	36.5 ± 4.2
Fat-free mass (kg) *	58.34 ± 4.39	57.18 ± 4.26 *	54.6 ± 3.0	55.3 ± 3.5
Fasting glucose (mmol/L) *	100.3 ± 2.8	92.1 ± 2.6 **	95.4 ± 3.7	94.8 ± 2.3
Fasting insulin (pmol/L)	108.3 ± 14.1	70.17 ± 7.8 **	124.4 ± 12.9	108.0 ± 12.3
GlucoseAUC (mmol/L/120 min) *	18,396 ± 1026	15,975 ± 1061 ‡	17,037 ± 1201	17,030 ± 1118
InsulinAUC (pmol/L/120 min)	67,144 ± 9590	54,624 ± 8655 *	76,575 ± 10,521	65,156 ± 10,306 *
HOMA-IR	4.95 ± 0.77	2.99 ± 0.38 **	4.97 ± 0.57	4.07 ± 0.51
RMR (kcal/d)	1523 ± 114	1483 ± 149	1376 ± 101	1542 ± 163 †
Fasting RQ (respiratory quotient)	0.75 ± 0.01	0.71 ± 0.06	0.73 ± 0.01	0.72 ± 0.06
Plasma ZAG (ng/mL)	290.37 ± 47.56	251.99 ± 53.24	258.28 ± 53.34	234.27 ± 47.36
Plasma HSL (ng/mL)	13.86 ± 6.46	15.43 ± 9.82	17.45 ± 12.65	20.61 ± 13.58

* *p* ≤ 0.05, † *p* ≤ 0.01, ** *p* ≤ 0.005, ‡ *p* ≤ 0.001; M = male, F = female; C = Caucasian, AA = African American; VO_2_max = maximal oxygen consumption; -AUC = area under the curve; RMR = resting metabolic rate. Data are mean ± SEM.

**Table 2:** Correlations between abdominal and gluteal ZAG, HSL and β3AR gene expression.

		AB ZAG	Glut ZAG	AB HSL	Glut HSL	AB B3AR	Glut B3AR
AB ZAG	r *p*	---	0.901 *** <0.001	0.652 *** 0.001	0.790 *** <0.001	0.577 ** 0.002	0.839 *** <0.001
Glut ZAG	r *p*	0.901 *** <0.001	---	0.487 * 0.012	0.783 *** <0.001	0.499 ** 0.010	0.746 *** <0.001
AB HSL	r *p*	0.652 *** 0.001	0.487 * 0.012	---	0.836 *** <0.001	0.487 * 0.012	0.395 * 0.046
Glut HSL	r *p*	0.790 *** <0.001	0.783 *** <0.001	0.836 *** <0.001	---	0.409 * 0.038	0.508 ** 0.008
AB B3AR	r *p*	0.577 ** 0.002	0.499 ** 0.010	0.487 * 0.012	409 * 0.038	---	
Glut B3AR	r *p*	0.839 *** <0.001	0.746 *** <0.001	0.395 * 0.046	0.508 ** 0.008		---

* *p* ≤ 0.05, ** *p* ≤ 0.01, *** *p* ≤ 0.001.

**Table 3:** Correlations between clinical characteristics and adipose tissue gene expression.

		AB ZAG	Glut ZAG	AB HSL	Glut HSL	AB B3AR	Glut B3AR	Plasma ZAG	Plasma HSL
Fasting Glucose	r *p*	−0.503 ** 0.010	−0.427 * 0.033	−0.665 *** <0.001	−0.673 *** <0.001	−0.434 * 0.030	−0.0352 0.085	•	•
Fasting Insulin	r *p*	•	•	•	•	•	•	•	0.487 * 0.019
Glucose AUC	r *p*	−0.485 * 0.016	−0.397 0.055	−0.538 ** 0.007	−0.555 ** 0.005	−0.473 * 0.020	−0.466 * 0.022	•	•
Insulin AUC	r *p*	•	−0.453 * 0.026	−0.425 * 0.038	−0.499 * 0.013	•	•	•	•
HOMA-IR	r *p*	•	•	−0.421 * 0.040	−0.442 * 0.030	•	•	•	0.461 * 0.027

* *p* ≤ 0.05, ** *p* ≤ 0.01, *** *p* ≤ 0.001, • no significant correlations. AB = abdominal adipose tissue; Glut = gluteal adipose tissue; AUC = area under the curve.

## Data Availability

Data may be available upon reasonable request.

## References

[1] Abdelaal M., le Roux C.W., Docherty N.G. (2017). Morbidity and mortality associated with obesity. Ann. Transl. Med..

[2] Choe S.S., Huh J.Y., Hwang I.J., Kim J.I., Kim J.B. (2016). Adipose Tissue Remodeling: Its Role in Energy Metabolism and Metabolic Disorders. Front. Endocrinol..

[3] Coelho M., Oliveira T., Fernandes R. (2013). Biochemistry of adipose tissue: An endocrine organ. Arch. Med. Sci..

[4] Burgi W., Schmid K. (1961). Preparation and properties of Zn-alpha 2-glycoprotein of normal human plasma. J. Biol. Chem..

[5] Bing C., Bao Y., Jenkins J., Sanders P., Manieri M., Cinti S., Tisdale M.J., Trayhurn P. (2004). Zinc-alpha2-glycoprotein, a lipid mobilizing factor, is expressed in adipocytes and is up-regulated in mice with cancer cachexia. Proc. Natl. Acad. Sci. USA.

[6] Bundred N.J., Miller W.R., Walker R.A. (1987). An immunohistochemical study of the tissue distribution of the breast cyst fluid protein, zinc alpha 2 glycoprotein. Histopathology.

[7] Russell S.T., Zimmerman T.P., Domin B.A., Tisdale M.J. (2004). Induction of lipolysis in vitro and loss of body fat in vivo by zinc-alpha2-glycoprotein. Biochim. Biophys. Acta.

[8] Gong F.Y., Zhang S.J., Deng J.Y., Zhu H.J., Pan H., Li N.S., Shi Y.F. (2009). Zinc-alpha2-glycoprotein is involved in regulation of body weight through inhibition of lipogenic enzymes in adipose tissue. Int. J. Obes..

[9] Ceperuelo-Mallafre V., Naf S., Escote X., Caubet E., Gomez J.M., Miranda M., Chacon M.R., Gonzalez-Clemente J.M., Gallart L., Gutierrez C. (2009). Circulating and adipose tissue gene expression of zinc-alpha2-glycoprotein in obesity: Its relationship with adipokine and lipolytic gene markers in subcutaneous and visceral fat. J. Clin. Endocrinol. Metab..

[10] Baskin A.S., Linderman J.D., Brychta R.J., McGehee S., Anflick-Chames E., Cero C., Johnson J.W., O'Mara A.E., Fletcher L.A., Leitner B.P. (2018). Regulation of Human Adipose Tissue Activation, Gallbladder Size, and Bile Acid Metabolism by a beta3-Adrenergic Receptor Agonist. Diabetes.

[11] Schena G., Caplan M.J. (2019). Everything You Always Wanted to Know about beta(3)-AR * (* But Were Afraid to Ask). Cells.

[12] Cero C., Lea H.J., Zhu K.Y., Shamsi F., Tseng Y.H., Cypess A.M. (2021). beta3-Adrenergic receptors regulate human brown/beige adipocyte lipolysis and thermogenesis. JCI Insight.

[13] Inokuma K., Okamatsu-Ogura Y., Omachi A., Matsushita Y., Kimura K., Yamashita H., Saito M. (2006). Indispensable role of mitochondrial UCP1 for antiobesity effect of beta3-adrenergic stimulation. Am. J. Physiol. Endocrinol. Metab..

[14] Kooijman S., van den Berg R., Ramkisoensing A., Boon M.R., Kuipers E.N., Loef M., Zonneveld T.C., Lucassen E.A., Sips H.C., Chatzispyrou I.A. (2015). Prolonged daily light exposure increases body fat mass through attenuation of brown adipose tissue activity. Proc. Natl. Acad. Sci. USA.

[15] van der Vaart J.I., Boon M.R., Houtkooper R.H. (2021). The Role of AMPK Signaling in Brown Adipose Tissue Activation. Cells.

[16] Russell S.T., Tisdale M.J. (2012). Role of beta-adrenergic receptors in the anti-obesity and anti-diabetic effects of zinc-alpha2-glycoprotien (ZAG). Biochim. Biophys. Acta.

[17] Russell S.T., Tisdale M.J. (2012). Role of beta-adrenergic receptors in the oral activity of zinc-alpha2-glycoprotein (ZAG). Endocrinology.

[18] Lass A., Zimmermann R., Oberer M., Zechner R. (2011). Lipolysis-A highly regulated multi-enzyme complex mediates the catabolism of cellular fat stores. Prog. Lipid Res..

[19] Fan G., Li Y., Ma F., Zhao R., Yang X. (2021). Zinc-alpha2-glycoprotein promotes skeletal muscle lipid metabolism in cold-stressed mice. Endocr. J..

[20] Althaher A.R. (2022). An Overview of Hormone-Sensitive Lipase (HSL). Sci. World J..

[21] Ge S., Ryan A.S. (2014). Zinc-alpha2-glycoprotein expression in adipose tissue of obese postmenopausal women before and after weight loss and exercise + weight loss. Metabolism.

[22] Lichtenstein A.H., Appel L.J., Brands M., Carnethon M., Daniels S., Franch H.A., Franklin B., Kris-Etherton P., Harris W.S., Howard B. (2006). Summary of American Heart Association Diet and Lifestyle Recommendations revision 2006. Arterioscler. Thromb. Vasc. Biol..

[23] Ryan A.S., Li G., McMillin S., Prior S.J., Blumenthal J.B., Mastella L. (2021). Pathways in Skeletal Muscle: Protein Signaling and Insulin Sensitivity after Exercise Training and Weight Loss Interventions in Middle-Aged and Older Adults. Cells.

[24] Ryan A.S., Nicklas B.J., Berman D.M. (2006). Aerobic exercise is necessary to improve glucose utilization with moderate weight loss in women. Obesity.

[25] Matthews D.R., Hosker J.P., Rudenski A.S., Naylor B.A., Treacher D.F., Turner R.C. (1985). Homeostasis model assessment: Insulin resistance and beta-cell function from fasting plasma glucose and insulin concentrations in man. Diabetologia.

[26] Ryan A.S., Novitskaya M., Treuth A.L. (2022). Predictive Equations Overestimate Resting Metabolic Rate in Survivors of Chronic Stroke. Arch. Phys. Med. Rehabil..

[27] Ryan A.S., Ge S., Blumenthal J.B., Serra M.C., Prior S.J., Goldberg A.P. (2014). Aerobic Exercise and Weight Loss Reduce Vascular Markers of Inflammation and Improve Insulin Sensitivity in Obese Women. J. Am. Geriatr. Soc..

[28] Ryan A.S., Li G., Blumenthal J.B., Ortmeyer H.K. (2013). Aerobic exercise + weight loss decreases skeletal muscle myostatin expression and improves insulin sensitivity in older adults. Obesity.

[29] Li G., Zhang H., Ryan A.S. (2020). Skeletal Muscle Angiopoietin-Like Protein 4 and Glucose Metabolism in Older Adults after Exercise and Weight Loss. Metabolites.

[30] Banaszak M., Gorna I., Przyslawski J. (2021). Zinc and the Innovative Zinc-alpha2-Glycoprotein Adipokine Play an Important Role in Lipid Metabolism: A Critical Review. Nutrients.

[31] Yang M., Liu R., Li S., Luo Y., Zhang Y., Zhang L., Liu D., Wang Y., Xiong Z., Boden G. (2013). Zinc-alpha2-glycoprotein is associated with insulin resistance in humans and is regulated by hyperglycemia, hyperinsulinemia, or liraglutide administration: Cross-sectional and interventional studies in normal subjects, insulin-resistant subjects, and subjects with newly diagnosed diabetes. Diabetes Care.

[32] Mracek T., Stephens N.A., Gao D., Bao Y., Ross J.A., Ryden M., Arner P., Trayhurn P., Fearon K.C., Bing C. (2011). Enhanced ZAG production by subcutaneous adipose tissue is linked to weight loss in gastrointestinal cancer patients. Br. J. Cancer.

[33] Yeaman S.J. (2004). Hormone-sensitive lipase--new roles for an old enzyme. Biochem. J..

[34] Jocken J.W., Langin D., Smit E., Saris W.H., Valle C., Hul G.B., Holm C., Arner P., Blaak E.E. (2007). Adipose triglyceride lipase and hormone-sensitive lipase protein expression is decreased in the obese insulin-resistant state. J. Clin. Endocrinol. Metab..

[35] Morigny P., Houssier M., Mairal A., Ghilain C., Mouisel E., Benhamed F., Masri B., Recazens E., Denechaud P.D., Tavernier G. (2019). Interaction between hormone-sensitive lipase and ChREBP in fat cells controls insulin sensitivity. Nat. Metab..

[36] Karki S., Farb M.G., Myers S., Apovian C., Hess D.T., Gokce N. (2015). Effect of Bariatric Weight Loss on the Adipose Lipolytic Transcriptome in Obese Humans. Mediat. Inflamm..

[37] Richelsen B., Pedersen S.B., Kristensen K., Borglum J.D., Norrelund H., Christiansen J.S., Jorgensen J.O. (2000). Regulation of lipoprotein lipase and hormone-sensitive lipase activity and gene expression in adipose and muscle tissue by growth hormone treatment during weight loss in obese patients. Metabolism.

[38] Ahn C., Ryan B.J., Schleh M.W., Varshney P., Ludzki A.C., Gillen J.B., Van Pelt D.W., Pitchford L.M., Howton S.M., Rode T. (2022). Exercise training remodels subcutaneous adipose tissue in adults with obesity even without weight loss. J. Physiol..

[39] Walhin J.P., Richardson J.D., Betts J.A., Thompson D. (2013). Exercise counteracts the effects of short-term overfeeding and reduced physical activity independent of energy imbalance in healthy young men. J. Physiol..

[40] Watt M.J., Heigenhauser G.J., Spriet L.L. (2003). Effects of dynamic exercise intensity on the activation of hormone-sensitive lipase in human skeletal muscle. J. Physiol..

[41] Walhin J.P., Dixon N.C., Betts J.A., Thompson D. (2016). The impact of exercise intensity on whole body and adipose tissue metabolism during energy restriction in sedentary overweight men and postmenopausal women. Physiol. Rep..

[42] Johnson W.J., Jang S.Y., Bernard D.W. (2000). Hormone sensitive lipase mRNA in both monocyte and macrophage forms of the human THP-1 cell line. Comp. Biochem. Physiol. B Biochem. Mol. Biol..

[43] Reue K., Cohen R.D., Schotz M.C. (1997). Evidence for hormone-sensitive lipase mRNA expression in human monocyte/macrophages. Arterioscler. Thromb. Vasc. Biol..

[44] O'Rourke L., Yeaman S.J., Shepherd P.R. (2001). Insulin and leptin acutely regulate cholesterol ester metabolism in macrophages by novel signaling pathways. Diabetes.

[45] Pandzic Jaksic V., Gizdic B., Miletic Z., Trutin-Ostovic K., Jaksic O. (2013). Association of monocyte CCR2 expression with obesity and insulin resistance in postmenopausal women. Clin. Investig. Med..

[46] Russell S.T., Tisdale M.J. (2010). Antidiabetic properties of zinc-alpha2-glycoprotein in ob/ob mice. Endocrinology.

[47] Robidoux J., Kumar N., Daniel K.W., Moukdar F., Cyr M., Medvedev A.V., Collins S. (2006). Maximal beta3-adrenergic regulation of lipolysis involves Src and epidermal growth factor receptor-dependent ERK1/2 activation. J. Biol. Chem..

[48] Greenberg A.S., Shen W.J., Muliro K., Patel S., Souza S.C., Roth R.A., Kraemer F.B. (2001). Stimulation of lipolysis and hormone-sensitive lipase via the extracellular signal-regulated kinase pathway. J. Biol. Chem..

[49] Russell S.T., Tisdale M.J. (2011). Studies on the antiobesity effect of zinc-alpha2-glycoprotein in the ob/ob mouse. Int. J. Obes..

[50] Ryan A.S., Katzel L.I., Prior S.J., McLenithan J.C., Goldberg A.P., Ortmeyer H.K. (2013). Aerobic Exercise Plus Weight Loss Improves Insulin Sensitivity and Increases Skeletal Muscle Glycogen Synthase Activity in Older Men. J. Gerontol. A Biol. Sci. Med. Sci..

[51] Ryan A.S., Ortmeyer H.K., Sorkin J.D. (2012). Exercise with calorie restriction improves insulin sensitivity and glycogen synthase activity in obese postmenopausal women with impaired glucose tolerance. Am. J. Physiol. Endocrinol. Metab..

[52] Goodpaster B.H., Kelley D.E., Wing R.R., Meier A., Thaete F.L. (1999). Effects of weight loss on regional fat distribution and insulin sensitivity in obesity. Diabetes.

[53] Abbasi F., Chang S.A., Chu J.W., Ciaraldi T.P., Lamendola C., McLaughlin T., Reaven G.M., Reaven P.D. (2006). Improvements in insulin resistance with weight loss, in contrast to rosiglitazone, are not associated with changes in plasma adiponectin or adiponectin multimeric complexes. Am. J. Physiol. Regul. Integr. Comp. Physiol..

[54] Berggren J.R., Hulver M.W., Dohm G.L., Houmard J.A. (2004). Weight loss and exercise: Implications for muscle lipid metabolism and insulin action. Med. Sci. Sports Exerc..

[55] Bogardus C., Ravussin E., Robbins D.C., Wolfe R.R., Horton E.S., Sims E.A. (1984). Effects of physical training and diet therapy on carbohydrate metabolism in patients with glucose intolerance and non-insulin-dependent diabetes mellitus. Diabetes.

